# β-hydroxybutyrate inhibits malignant phenotypes of prostate cancer cells through β-hydroxybutyrylation of indoleacetamide-N-methyltransferase

**DOI:** 10.1186/s12935-024-03277-6

**Published:** 2024-03-30

**Authors:** Yifan Zhang, Yunlong Li

**Affiliations:** Department of Urology, The First Affiliated Hospital of Zhengzhou University, No. 1 Jianshe East Road, Erqi District, Zhengzhou, Henan, Henan 450000 China

**Keywords:** β-hydroxybutyrate, Prostate cancer, Stemness, INMT, Kbhb

## Abstract

**Background:**

Prostate cancer (PCa) is one of the most prevalent cancers in men and is associated with high mortality and disability rates. β-hydroxybutyrate (BHB), a ketone body, has received increasing attention for its role in cancer. However, its role in PCa remains unclear. This study aimed to explore the mechanism and feasibility of BHB as a treatment alternative for PCa.

**Methods:**

Colony formation assay, flow cytometry, western blot assay, and transwell assays were performed to determine the effect of BHB on the proliferation and metastasis of PCa cells. Tumor sphere formation and aldehyde dehydrogenase assays were used to identify the impact of BHB or indoleacetamide-N-methyltransferase (INMT) on the stemness of PCa cells. N6-methyladenosine (m6A)–meRIP real-time reverse transcription polymerase chain reaction and dual luciferase assays were conducted to confirm INMT upregulation via the METTL3–m6A pathway. Co-IP assay was used to detect the epigenetic modification of INMT by BHB-mediated β-hydroxybutyrylation (kbhb) and screen enzymes that regulate INMT kbhb. Mouse xenograft experiments demonstrated the antitumor effects of BHB in vivo.

**Results:**

BHB can inhibit the proliferation, migration, and invasion of PCa cells by suppressing their stemness. Mechanistically, INMT, whose expression is upregulated by the METTL3–m6A pathway, was demonstrated to be an oncogenic gene that promotes the stem-like characteristics of PCa cells. BHB can suppress the malignant phenotypes of PCa by kbhb of INMT, which in turn inhibits INMT expression.

**Conclusions:**

Our findings indicate a role of BHB in PCa metabolic therapy, thereby suggesting an epigenetic therapeutic strategy to target INMT in aggressive PCa.

**Trial registration:**

Not applicable.

**Supplementary Information:**

The online version contains supplementary material available at 10.1186/s12935-024-03277-6.

## Background

Prostate cancer (PCa) is the second most frequent malignancy in males, is the fifth leading cause of cancer-related deaths in both developed and developing countries, and accounts for a significant proportion of the health burden worldwide [1]. Currently, no long-lasting and effective therapies exist for PCa. PCa treatment via androgen deprivation therapy and AR antagonists (castration), along with the emergence of PCa with AR mutations or lack of AR expressio, has been shown to be effective only in the short term [1]. Other PCA treatment options include surgery, chemotherapy, radiation therapy, and immunotherapy [2–4]. However, with the advent of drug resistance, cancer metastasis, and vigorous weakening of immunity, the effectiveness of these treatment options has decreased. Dietary therapy (or metabolic therapy) using ketogenic diets (KD) is becoming an alternative or complementary approach to cancer treatment [5]. Most cancer cells are characterized by a lack of mitochondrial enzymes capable of metabolizing ketone bodies and producing ATP.

In contrast, normal cells can use ketone bodies instead of sugar as fuel [6]. KD combats cancer-induced cachexia and causes minimal side effects [7]. KD disrupts metabolism and suppresses the “Warburg effect” on which tumor growth depends [8]. However, the effects of KD on PCa remain unclear. Hence, this study mainly aimed to investigate whether ketone bodies have an anticancer effect on PCa.

β-hydroxybutyrate (BHB), the primary ketone body, is predominantly formed in the liver and formed by the degradation of fatty acids via β-oxidation induced by acetyl coenzyme A. BHB is traditionally considered a normal fuel to support respiration and serve as an alternative energy resource for the brain and heart during fasting and prolonged exercise [9]. Increasing evidence indicates a strong relationship between BHB and cancer. Several studies have indicated that BHB can suppress the growth and metastasis of glioma, neuroblastoma, pancreatic cancer, and colorectal cancer [5, 10–14] and even enhance the anticancer effects of cisplatin and PD-1 blockade [15, 16]. In contrast, some studies have demonstrated that BHB promotes the growth and survival of cancer cells [17, 18], whereas another study showed no impact on growth [19]. The BHB paradox and the effect of BHB on PCa warrant further in-depth exploration.

Cancer stem cells (CSCs) represent a small number of undifferentiated cells in cancerous tissues. The presence of CSCs with properties of tumor dissemination and metastasis promotion can significantly affect disease progression and clinical management and is a major cause of metastasis and cancer recurrence [20]. PCa involves highly heterogeneous cells [21]. CSCs residing in PCa tissue may constitute an important cause of the development and recurrence of PCa as well as the development of androgen-independent and refractory phenotypes [22, 23]. However, cancer cells can switch between stem and differentiated states in response to treatment or microenvironmental changes [24, 25]. Therefore, the development of specific anticancer drugs that can eradicate CSCs or induce their differentiation is an innovative therapeutic strategy for PCa.

Indoleacetamide-N-methyltransferase (INMT) acts as a methyltransferase, both as an S-methyltransferase for thymidine and N-methyltransferase for catalyzing the N-methylation of indoles such as tryptamine [26, 27]. The colocalization of INMT with the sigma-1 receptor in primate spinal motor neurons revealed that it may be a target for treating schizophrenia and amyotrophic lateral sclerosis [28, 29]. In addition to its association with schizophrenia, the role of INMT in cancer is gradually being recognized. However, whether INMT is an oncogene or cancer suppressor remains controversial in the recently published studies on PCa [30, 31]. The effect of INMT on PCa and its molecular mechanisms warrant further exploration. β-hydroxybutyrylation (kbhb) is an emerging post-translational modification (PTM) induced by BHB. So far, kbhb has been described in histones and nonhistones [32, 33]. Mass spectrometry analysis in a previous study showed that INMT might be β-hydroxybutyrylated [34]. However, the mechanism by which the kbhb of INMT affects its role in cancer has not yet been reported, and our study investigated for the first time the role of INMT kbhb in PCa.

Herein, we aimed to demonstrate that BHB acts as an important ketone body to inhibit the proliferation, migration, and invasion of PCa cells by suppressing their stem-like properties. INMT, whose expression is upregulated by the METTL3–N6-methyladenosine (m6A) axis, is an oncogene in PCa that promotes the stemness of PCa cells through SOX2. BHB inhibits the malignant phenotypes of PCa via kbhb of INMT. Therefore, this study may provide potential metabolic therapy and molecular targets for refractory PCa.

## Methods

### Chemicals and antibodies

The chemical reagents and working concentrations used were as follows: BHB (for cells: 0, 15, and 25 mM; for mice: 100 mg/kg; Sigma-Aldrich, cat. 52,017) and p300 inhibitor A485 (for cells: 2.5 µM; for mice: 30 mg/kg; Sigma-Aldrich, cat. SML2192). The antibodies used were as follows: anti-SLUG (Abcam, cat. ab27568), anti-TWIST1 (Cell Signaling Technology, cat. #90,445), anti-E-cadherin (Abcam, cat. ab231303), anti-vimentin (Sigma-Aldrich, cat. V6630), anti-snail (Sigma-Aldrich, cat. SAB5700806), anti-ZEB1 (Abcam, cat. ab276129), anti-ZEB2 (Abcam, cat. ab191364), anti-GAPDH (Abcam, cat. ab8245), anti-SOX2 (Cell Signaling Technology, cat. #14,962), anti-BMI1 (Abcam, cat. ab269678), anti-CD133 (Abcam, cat. ab284389), anti-KLF4 (Abcam, cat. ab129473), anti-β actin (Cell Signaling Technology, cat. #3700), anti-INMT (Abcam, cat. ab181854), anti-METTL3 (Abcam, cat. ab195352), anti-METTL14 (Abcam, ab220030), anti-ALKBH5 (Abcam, cat. ab195376), anti-FTO (Abcam, cat. ab280081), anti-m6A (Invitrogen, cat. MA5-33030), anti-Ki67 (Abcam, cat. ab15580), and anti-pan BHB-lysine (BHB-K) (PTM BioLabs, China, cat. #PTM-1201RM).

### Cell culture

PC3 and LNCaP were purchased from Procell (Wuhan, China), DU145, RWPE-1, and human embryonic kidney 293E (HEK293E) cells from ATCC were gifted by Dr. Qi Li (The First Affiliated Hospital of Zhengzhou University, Zhengzhou, China) and cultured in a humidified environment at 37 °C under 5% CO_2_ using their respective media. RWPE-1 cells were maintained in keratinocyte SFM (1×) (Invitrogen, cat. 17,005,042). PC3 and LNCaP cells were cultured in RPMI 1640 supplemented with 15% fetal bovine serum (FBS). DU145 and HEK293E cells were cultured in Dulbecco’s modified Eagle’s medium (DMEM) with 15% FBS. LNCaP and PC3 cells stably overexpressing control and INMT vectors were cultured in RPMI 1640 supplemented with 15% FBS and hygromycin (150 µg/ml) [30, 31]. LNCaP and PC3 cells stably expressing control shRNA (Sigma-Aldrich, cat. SHC016) and INMT shRNA (Sigma-Aldrich, cat. EHU138831) were cultured in RPMI 1640 supplemented with 15% FBS and puromycin (15 µg/ml) [31].

### Transfection, plasmids, and siRNA

Lipofectamine 3000 (Invitrogen, cat. L3000015) was used to transfect plasmids, whereas Lipofectamine RNAiMAX (Invitrogen, cat. 13,778,030) was used to transfect siRNAs, according to the manufacturer’s instructions of the corresponding kits.

pGL3-INMT-WT, pGL3-INMT-mut, and pGL3-SOX2 plasmids were constructed by inserting the INMT 3′-UTR with wild-type or mutant m6A sites and SOX2 promoters (− 2546/+544) into pGL3 luciferase reporter plasmids (Promega, cat. E1751).

pFlag–METTL3, pFlag–INMT, pFlag–HDAC1, and pFlag–HDAC2 plasmids were constructed by cloning polymerase chain reaction (PCR)-amplified cDNAs of human METTL3, INMT, HDAC1, and HDAC2 into the pFlag–CMV2 expression vectors (Sigma-Aldrich, cat. E7033).

The sequences of METTL3-specific siRNAs were as follows: #1: 5′-CUGCAAGUAUGUUCACUAUGA-3′ and #2: 5′-AGGAGCCAAGAAAAAUCAA-3′).

### Cell viability assay

Cell viability was assessed via the CCK8 assay using a CCK8 kit (Abcam, cat. ab228554). Cells (1 × 10^4^/well) inoculated in a 96-well plate were treated with varying concentrations of BHB for 48 h. Then, 20 µl of CCK-8 solution was added to the corresponding wells and incubated for 2 h. Absorbance values at 450 nm were measured using a 96-well plate reader. The concentration of the drug that induced 50% of cell growth inhibition (IC50) was determined.

### Colony formation assay

Colony formation assay was performed to assess the cell proliferation capacity. LNCaP and PC3 cells (2 × 10^4^/well) were inoculated into 6-well plates and treated with or without 15 mM BHB for 48 h. After washing the cells with PBS, the liquid in each well was replaced with fresh medium without drugs. Two weeks later, using methanol fixation and crystal violet staining, the number of colonies containing > 50 cells were counted using an optical microscope.

### Cell cycle analysis

LNCaP and PC3 cells were inoculated into 6-well plates at a concentration of 2 × 10^5^/well and treated with or without 15 mM BHB for 48 h. After washing with PBS and fixing overnight at 4 °C with 75% ethanol, the fixed cells were incubated for 30 min under dark conditions with 1 ml of PBS containing propyl iodide (PI) (100 µg/ml) and RNase (50 µg/ml). Flow cytometry was performed to analyze cell cycle distribution. DNA histograms were constructed to indicate the proportion of cells in different cell cycle phases, such as G0/G1, S, and G2/M phases.

### Cell apoptosis analysis

Flow cytometric analysis was performed using FITC/Annexin V apoptosis-detecting kits (BD pharmingen, cat. NO 556,547) to determine the apoptosis rate following BHB treatment. After treatment with 0, 15, or 25 mM BHB for 48 h, 1 × 10^6^ cells (LNCaP, PC3, or DU145 cells) were collected and cleaned twice with ice-cold PBS. After centrifugation at 500 ×*g* for 5 min, the cells were added to 100 µl of binding buffer containing 5 µl of PI and 5 µl of Annexin V-FITC, and the mixture was incubated for 15 min under dark conditions. The apoptosis rate of stained cells was immediately measured at 488 nm using BD FACSAria™ II cell sorters (BD Biosciences, California, USA).

### Transwell cell migration and invasion assay

Transwell assay was performed to determine the cell migration and invasive capacity. For migration assays, treated cells were harvested and diluted with serum-free DMEM (1 × 10^5^ cells/ml). Then, 100 ml of cell suspension was inoculated into the small chamber above the transwell insert (Corning, cat. CLS3464; pore size, 8 μm), whereas the bottom chambers were filled with RPMI-1640 containing 20% FBS, serving as a chemoattractant. For invasive assays, the above chambers were precoated with Matrigel (Millipore, cat. E1270); the other steps were the same as the migration assays. The cells were cultured at 37 °C for 24 h under 5% CO_2_. Migrated and invaded cells at the bottom of the filter were fixed with 4% paraformaldehyde and stained with 0.5% crystal violet (Sigma-Aldrich, cat. C0775) for 20 min. Cells were observed and counted under optical microscopes. Five regions were randomly selected to count the number of cells.

### Western blot assay

Whole-cell lysates were prepared using highly active radioimmunoprecipitative assay (RIPA) buffer (Beyotime, cat. P0013B). Protein concentrations were determined via an enhanced protein concentration assay kit (Beyotime, China, P0010S). Whole-cell proteins in the supernatant were electrophoresed on 10% SDS–PAGE gel and then transferred onto the PVDF membrane (Millipore, cat. ISEQ00010). These membranes were subjected to incubation with the corresponding primary and HRP-coupled secondary antibodies. The bands were visualized using BeyoECL plus kit (Beyotime, cat. P0018M).

### Real-time reverse transcription PCR (RT–PCR)

Total cellular RNA was purified using Trizol reagent (Beyotime, cat. R0016). Isolated RNA was reverse transcribed using Maxima H Minus first strand cDNA synthesis kits (Thermo Scientific, cat. #K1682). Quantitative real-time PCR (qPCR) was performed using SYBR Green (Takara, cat. #RR820A) via ABI-7500 real-time PCR systems (Applied Biosystems, USA). The specific primers used in this study are listed in Supplemental Table S1. The relative expression levels of target genes were measured using the 2^−ΔΔCT^ method.

### Tumor sphere formation assay

The treated cells were prepared as single-cell suspensions and seeded into ultralow adherence 96-well plates at a density of 800 cells/well containing sphere-forming medium supplemented with serum-free DMEM/F-12 (Invitrogen, cat. 12,634,028), 2% B27 (Invitrogen, cat. 17,504,044), 25 ng/ml epidermal growth factor (EGF; Sigma-Aldrich, cat. E9644), and 25 ng/ml fibroblast growth factor (FGF; Gibco, cat. 13256-029). The cells were incubated at 37 °C for 7–15 days under 5% CO_2_ and 95% humidity. The number of spheroids with diameters of > 25 or 75 μm was calculated.

### Aldehyde dehydrogenase (ALDH) assay

The stem-like tumor cells with highly active ALDH were identified using ALDEFLUOR kits (Stem Cell Technologies, cat. #01700), according to the manufacturer’s instructions. Briefly, 1 × 10^5^ cells were incubated with ALDEFLUOR assay buffer supplemented with ALDH substrate. An aliquot of cells exposed to ALDEFLUOR assay buffer was treated with a specialized ALDH inhibitor (diethylaminobenzaldehyde, DEAB) under the same conditions; this was set as a negative control. After incubation at 37 °C for 40 min, the fluorescence intensity was measured via FACS analysis.

### m6A meRIP qRT–PCR

The m6A modification of INMT was measured via MeRIP assays using m6A MeRIP kits (Millipore, cat. 17-10499), according to the manufacturer’s instructions. Briefly, 300 µg of total isolated RNA was chemically fragmented into approximate lengths of 100 nucleotides and then immunoprecipitated with a monoclonal antibody against m6A (Invitrogen, cat. MA5-33030) and prewashed protein A/G Dyna beads (Thermo Scientific, cat. 88,803). The RNAs undergoing m6A modification were eluted with N6-methyladenosine-5′-monophosphate sodium salt (6.7 mM) and extracted using RNeasy kit (Qiagen, cat. 74,004). Both immunoprecipitated and input samples were subjected to qPCR.

### Dual luciferase reporter assays

To confirm whether INMT mRNA undergoes METTL3-dependent m6A modifications, pGL3-INMT-WT or pGL3-INMT-mut plasmids were constructed by inserting INMT mRNA 3′-UTRs with wild-type or mutant m6A sites into a region downstream of pGL3 luciferase reporter vector. The cells inoculated into a 12-well plate were transiently transfected with pGL3-INMT-WT or pGL3-INMT-mut, renilla luciferase reporter vectors (pRL-TK), and METTL3 siRNA. After 48 h of transfection, firefly luciferase activity was assessed using a dual luciferase reporter kit (Promega, cat. E1910). Measurement of renilla luciferase activity was used as a control for determining transfection efficiency.

To confirm the effect of INMT on SOX2 promoter activity. The pGL3-SOX2 promoter plasmids were constructed by inserting the SOX2 promoter (− 2546/+544) into a region downstream of pGL3 luciferase reporter vectors (Promega, cat. E1751). Cells stably expressing INMT or control vectors were placed in 12-well plates and transiently transfected with pGL3-SOX2 promoter and renilla luciferase reporter vectors (pRL-TK). The subsequent steps were the same as those described above.

### Protein purification

After transfection of 293T cells with Flag–INMT plasmids for 48 h, the cells were collected and lysed with BC500 solution (500 mM NaCl, 20% glycerol, 0.5% Triton X-100, and 20 mM Tris-HCl; pH 7.3) under sonication. The cell lysates were coincubated overnight at 4 °C with anti-Flag M2 magnetic beads. After washing the bead complexes with BC100 buffer (100 mM NaCl, 20% glycerol, 0.1% Triton X-100, and 20 mM Tris-HCl; pH 7.3), a competitive elution procedure with Flag peptide (Sigma-Aldrich, cat. F4799) was performed in BC100 buffer for protein purification.

### Evaluation of kbhb of INMT

Evaluation of kbhb of exogenous INMT proteins was performed as follows. First, 293T cells were transfected with Flag–INMT plasmids and then treated with or without 15 mM BHB for 24 h. The Flag–INMT fusion proteins were purified according to the method described previously in this study. Next, the purified proteins were subjected to western blot assay with anti-INMT or anti-pan BHB-K antibodies.

Evaluation of kbhb of endogenous INMT proteins was performed in LNCaP, PC3, and DU145 cells treated with or without 15 mM BHB for 24 h. The cells were first lysed with RIPA buffer and fragmented ultrasonically. Identification of BHB-K proteins was performed via immunoprecipitation (IP) with BHB-K antibodies, and INMT was detected via western blot assay using anti-INMT antibody.

Similarly, INMT kbhb was evaluated in LNCaP cells transfected with Flag–HDAC1 or Flag–HDAC2 plasmids and treated with 15 mM BHB or 2.5 µM p300 inhibitor A485 for 24 h. The subsequent steps were identical to those described above.

### Mouse xenograft assays

Male BALB/c nude mice (5 weeks old, weight 18–22 g) were obtained from the Animal Experiment Center of Zhengzhou University (Zhengzhou, China). They were maintained under standard conditions. Overall, 5 × 10^6^ LNCaP cells were diluted with 200 µl of RPMI 1640 medium, prepared as a mixture with Matrigel (Corning, cat. 354,234), and inoculated subcutaneously into the flanks of male nude mice. Tumor size was measured every 3 days using calipers, and the tumor volume was calculated using the following formula: L (longest diameter) × W (shortest diameter)^2^ × 0.5. Each mouse’s body weight and living behavior were monitored for overall health status. The mice were euthanized at the end of the studies. The xenograft tumors were removed, weighed, and fixed. Mice were randomly divided into four groups for drug treatments when the measured tumor volume reached 150–200 mm [3]. They (seven mice for each group) received 0.9% saline as a negative control or 100 mg/kg BHB or 30 mg/kg p300 inhibitor A485 as treatment. The drugs were injected intraperitoneally daily for up to 30 days. Each procedure was approved by the Animal Care and Use Committee of the First Affiliated Hospital of Zhengzhou University. The HE and immunohistochemistry staining for Ki-67 were performed as reported previously [35]. Mice were injected with LNCaP cells stably transfected with luciferase vector via tail vein for establishing an in vivo bioluminescence imaging model of bone metastasis, and the detailed steps were performed according to the previously reported method [36].

### Statistical analysis

All data were expressed as the mean ± standard deviation (SD). Statistical analysis was performed via GraphPad Prism 8 software. Comparisons of parameters between two groups were calculated using paired two-tailed Student’s t-tests. *P*-values of < 0.05 were considered to indicate statistical significance.

## Results

### BHB significantly suppresses the proliferation, migration, and invasion of PCa cells

**Fig. 1:**
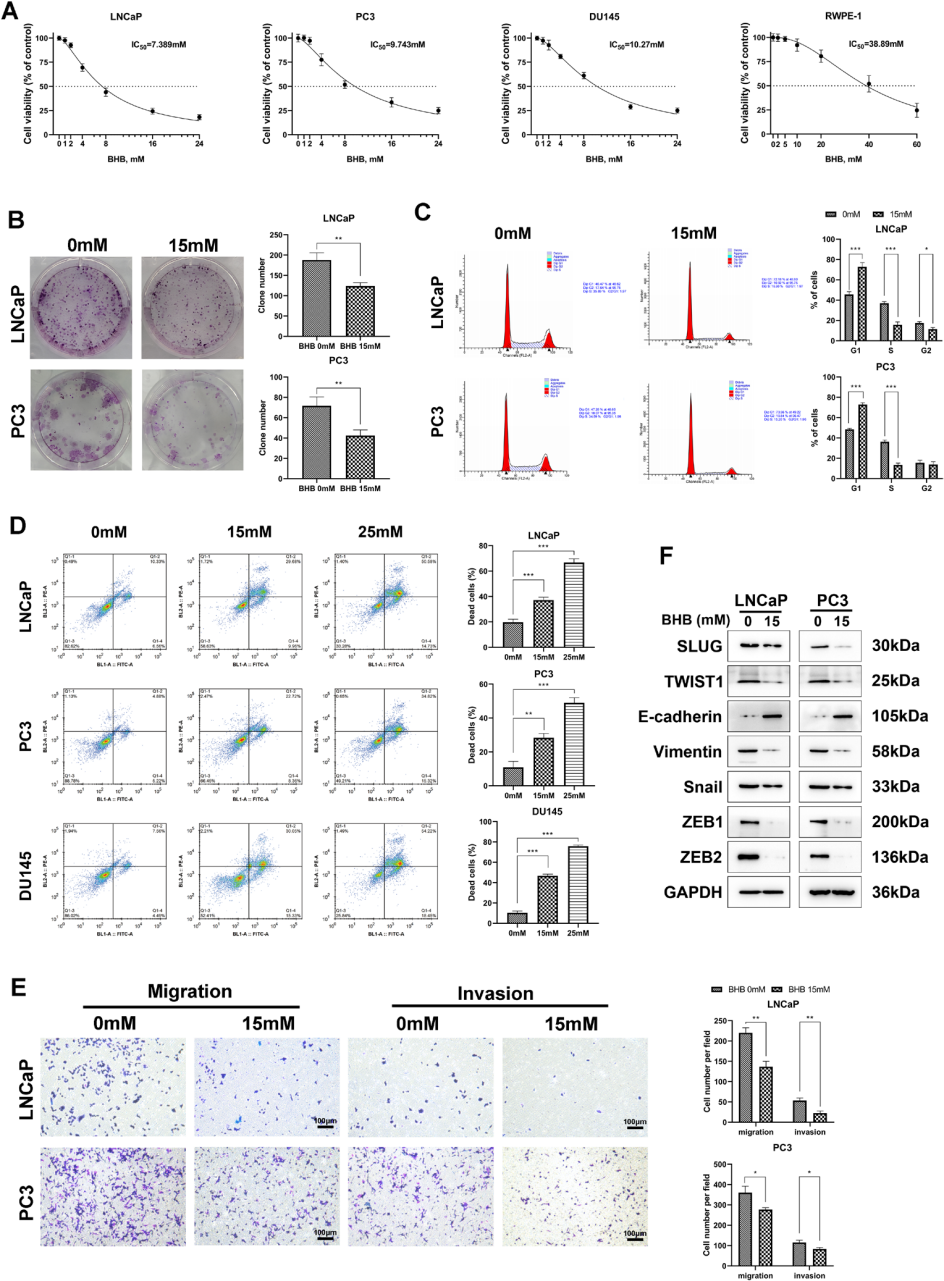
BHB significantly suppresses the proliferation, migration, and invasion of PCa cells. **A**, LNCaP, PC3, DU145, and RWPE-1 cells treated with various concentrations of BHB for 48 h were subjected to CCK8 assay. **B**, Colony formation assays were used to assess the in vitro tumor growth ability of cells treated with 0 or 15 mM BHB. The representative images and statistical charts are presented. **C**, Cell cycle assays were performed via flow cytometry in cells treated with or without 15 mM BHB. The ratio of cells distributed in three different phases was calculated. **D**, LNCaP, PC3, and DU145 cells were exposed to the corresponding concentrations of BHB for 48 h. The apoptotic rates were analyzed via flow cytometry using Annexin V-FITC/PI double staining methods. The representative images and statistical charts are shown. **E**, Migrative and Matrigel invasive ability of cells treated with or without 15mM BHB was tested via transwell assays. Representative images of migrating and invading cells on polycarbonate transwell membranes and statistical plots are shown. **F**, Western blot assays were used to detect the expression of representative epithelial-to-mesenchymal transition (EMT)-associated proteins in cells treated with or without BHB. All data were obtained by performing at least three replicate experiments and expressed as mean ± standard deviation (SD). Paired and two-tailed Student’s t-tests were performed, and significant differences were designated as *p < 0.05, **p < 0.01, and ***p < 0.001

The effects of BHB on the growth characteristics of normal prostate cell lines (RWPE-1) and PCa cell lines (PC3, LNCaP, and DU145) were initially analyzed. The cells were incubated for 48 h with increasing concentrations of BHB, and CCK8 assays were performed to analyze cell viability. We revealed that BHB suppressed the growth of LNCaP, PC3, and DU145 cells with inhibitory concentrations (IC_50_) of 7.389, 9.743, and 10.27 mM, respectively (Fig. 1A). In contrast, the IC_50_ of RWPE-1 cells was 38.89 mM, indicating that BHB induces little toxicity to normal prostate cells at concentrations that inhibit the growth of PCa cells (Fig. 1A).

The long-term proliferative capacity of malignant tumor cells was assessed via the colony formation assay using LNCaP and PC3 cells. When these cells were treated with 15 mM BHB for 48 h, their proliferative capacity was significantly suppressed, as evidenced by a decrease in the number of cells and a reduction in the size of cell colonies (Fig. 1B).

To explore the effects of BHB on cell cycle distribution, LNCaP and PC3 cells were treated with 15 mM BHB for 48 h, after which the DNA content of cells in different phases was analyzed via flow cytometry. The results showed that BHB-treated cells displayed a larger G0/G1-phase population and a smaller S-phase population, suggesting that BHB causes cell cycle arrest in tumor cells in the G0/G1 phase (Fig. 1C).

The proapoptotic effects of BHB were evaluated in BHB-treated cells via the PI and Annexin V double staining method, and the samples were analyzed via flow cytometry. We found that BHB induced apoptosis in LNCaP, PC3, and DU145 cells in a dose-dependent manner (Fig. 1D).

The migrative and invasive capacity of LNCaP and PC3 cells was analyzed via transwell assays with or without BHB treatment. All results indicated that cells treated with 15 mM BHB had a significantly reduced ability to migrate through the membranes into the lower lumen compared with untreated cells. In addition, the capacity of cells treated with BHB to invade the Matrigel precoated membranes and migrate to the lower lumen was significantly reduced compared with that of untreated cells (Fig. 1E).

Epithelial-to-mesenchymal transition (EMT) of tumor cells can drive tumor migration and invasion [37]. Thus, the effects of BHB on the expression of EMT markers were assessed via western blot analysis. We revealed that after BHB treatment, the expression of E-cadherin was strongly upregulated, whereas the levels of various mesenchymal markers, such as SLUG, TWIST1, vimentin, snail, ZEB1, and ZEB2, were decreased in LNCaP and PC3 cells (Fig. 1F).

### BHB inhibits the stem-like properties of PCa cells

**Fig. 2:**
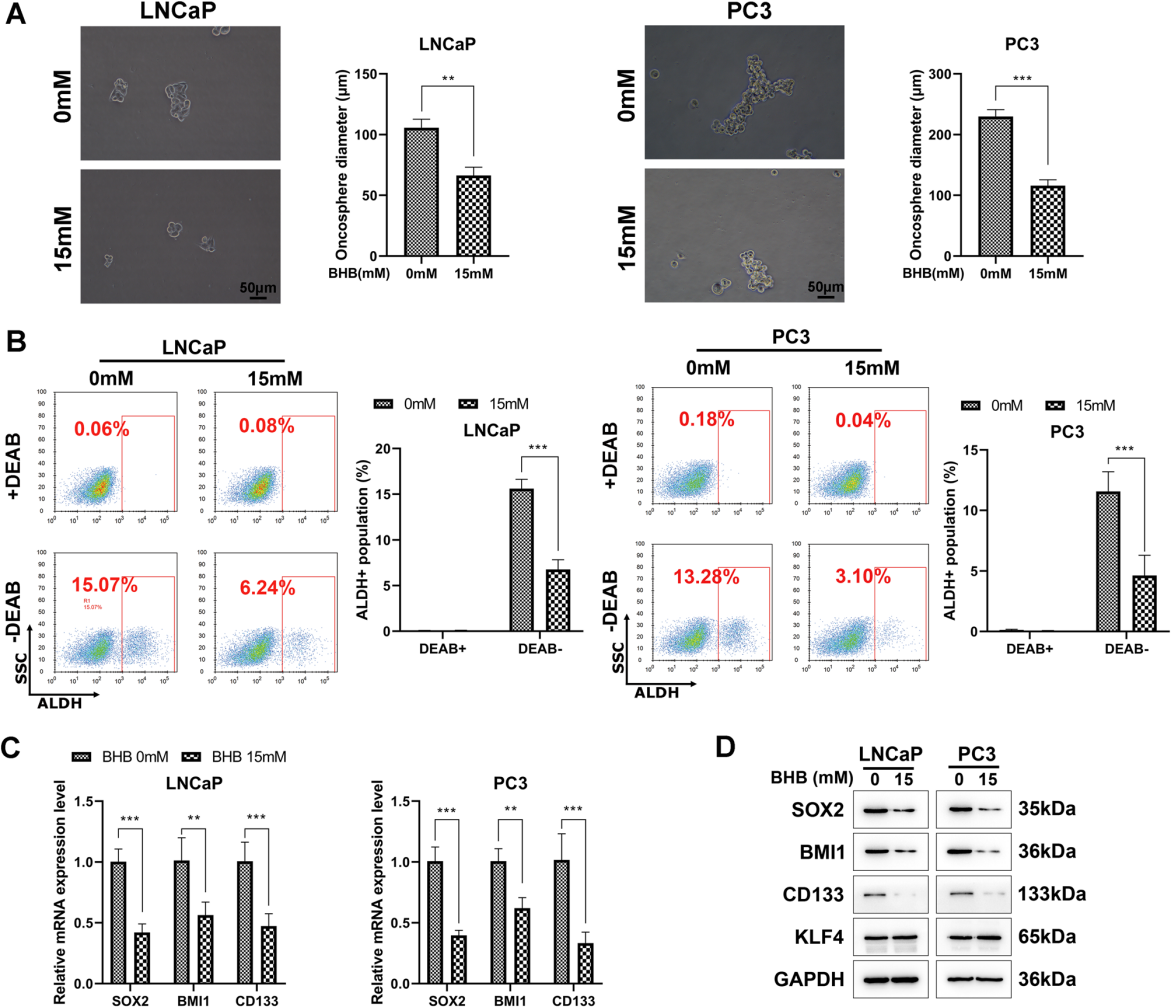
BHB inhibits the stem-like properties of PCa cells. **A**, The suppressive effects of BHB on PCa stem cells (CSCs) were measured via tumor sphere formation assays. Cells treated with or without 15 mM BHB were prepared as single-cell suspensions and seeded into ultralow attachment 96-well plates at a density of 500 cells/well containing sphere-forming medium. Cells were cultured continuously for 7–14 days, and the spheres with a diameter of > 25 or 75 μm were counted. **B**, The suppressive effects of BHB on prostate CSCs were measured via ALDH assays; after BHB treatment for 48 h, ALDH substrate was added and incubated for another 30 min. Then, flow cytometry was performed to measure the percentage of ALDH-positive cells. DEAB-quenched ALDH fluorescence was considered a negative control. **C**, The mRNA levels of prostate CSC-associated markers were examined via real-time qPCR in cells treated with or without BHB for 48 h. **D**, Protein levels of prostate CSC-associated markers were measured via western blot assay in cells treated with or without BHB for 48 h. Experiments were performed in triplicate, and data were expressed as the mean ± standard deviation (SD). Paired and two-tailed Student’s t-tests were performed, and significant differences were designated as *p < 0.05, **p < 0.01, and ***p < 0.001

Tumor sphere formation is a stem-like feature indicating the tumor-initiating capacity. As expected, BHB-treated LNCaP and PC3 cells formed fewer tumor spheres than untreated cells within 7 days (Fig. 2A). Subsequently, ALDH activity was analyzed via ALDEFLUOR assays based on a previous finding that increased ALDH activity is positively associated with CSC characteristics and poor prognosis in many cancers, especially PCa [38]. The proportion of ALDH-positive cells in LNCaP and PC3 cells treated with BHB was substantially lower than that in untreated cells (Fig. 2B). In addition, qPCR revealed that BHB treatment inhibited the mRNA expression levels of several stemness-related factors, such as SOX2, BMI1, and CD133, in LNCaP and PC3 cells (Fig. 2C). Consistently, western blot analysis indicated that the proteins levels of SOX2, BMI1, and CD133 were significantly decreased after BHB treatment, whereas the protein level of KLF4 remained unchanged after BHB treatment (Fig. 2D). Altogether, these results suggest that BHB can suppress the stem-like properties of PCa cells.

### INMT promotes the stemness of PCa cells through SOX2

**Fig. 3:**
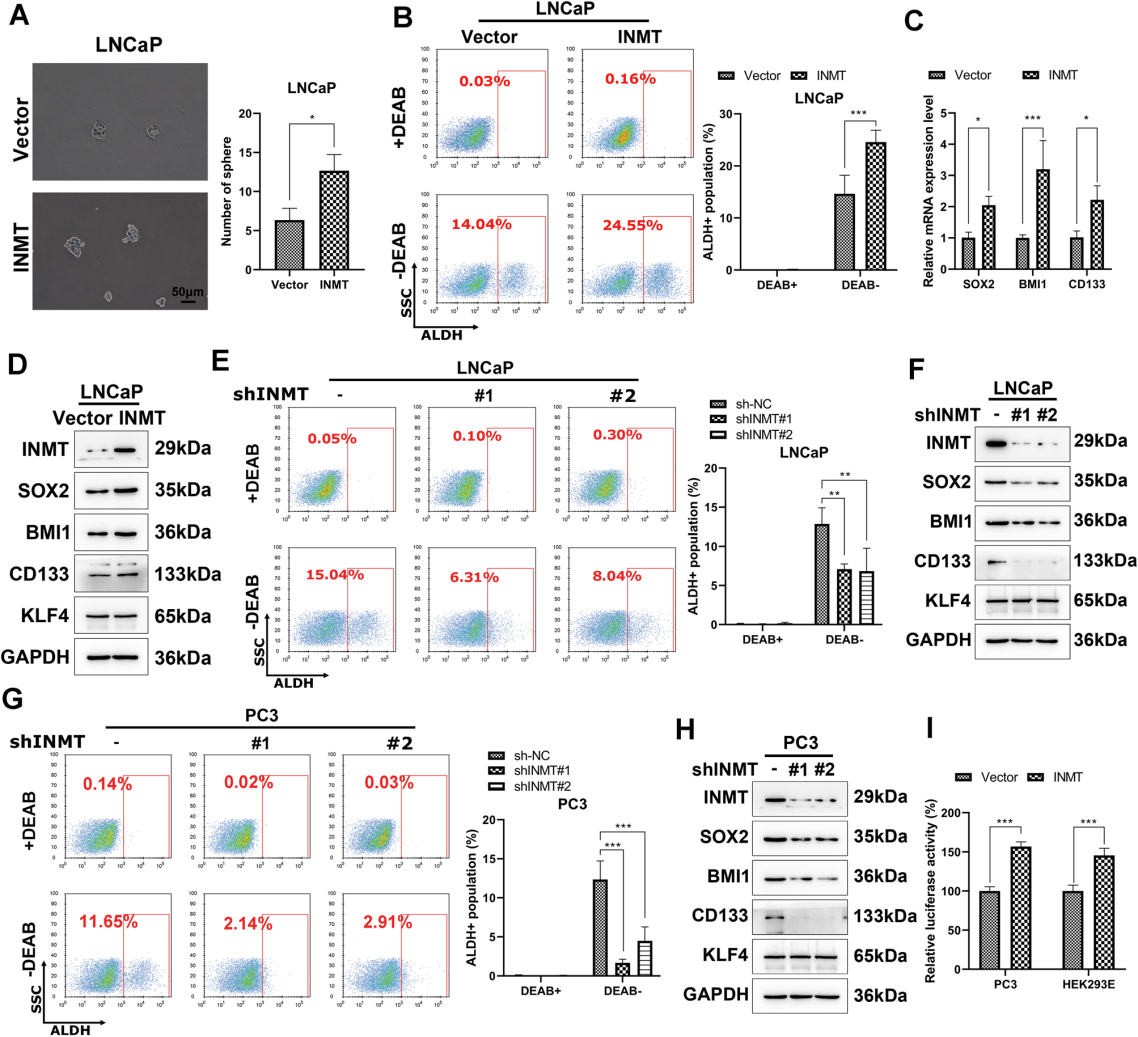
INMT promotes the stemness of PCa cells through SOX2. **A**, The effects of INMT overexpression on prostate CSCs were detected via tumor sphere formation assays. LNCaP cells were transfected with INMT or vector plasmids and tested for their tumor sphere formation ability. **B**, The effects of overexpressed INMT on prostate CSCs were determined via the ALDH assay. LNCaP cells transfected with INMT or vector plasmids were subjected to flow cytometry to measure the proportion of ALDH-positive cells. **C**, The mRNA levels of prostate CSC-associated markers were examined via real-time qPCR after *INMT* overexpression in LNCaP cells. **D**, Protein levels of prostate CSC-associated markers were measured via western blotting after *INMT* overexpression in LNCaP cells. **E** and **G**, The effect of *INMT* knockdown on prostate CSCs was determined by performing ALDH assay in LNCaP and PC3 cells. **F** and **H**, The protein levels of prostate CSC-associated markers were measured via western blot assay after *INMT* knockdown in LNCaP and PC3 cells. **I**, The effects of INMT on SOX2 promoter activity was detected using dual luciferase reporter. Cells were cotransfected with an INMT expression plasmid and a SOX2 promoter reporter plasmid. Firefly luciferase activity, which represents SOX2 promoter activity, was measured and normalized to renilla luciferase activity. Experiments were conducted in triplicate, and data were expressed as the mean ± standard deviation (SD). Paired and two-tailed Student’s t-tests were performed, and significant differences were designated as *p < 0.05, **p < 0.01, and ***p < 0.001

A previous study revealed that the PTM of INMT could be regulated by BHB; hence we speculated INMT as a therapeutic target of BHB on PCa. To prove this speculation, we next explore the involvement of INMT and the related mechanism in PCa. We firstly determined how INMT promotes the stemness of PCa cells by detecting tumor cell sphere formation. LNCaP cells with overexpressed *INMT* showed the formation of more tumor spheroids within 7 days than vector-transfected cells (Fig. 3A). When analyzing ALDH activity, which represents the stemness capacity, using ALDFHLUOR assays, we revealed that the proportion of ALDH-positive cells was significantly higher in INMT-overexpressed LNCaP cells than in vector-transfected cells (Fig. 3B). Real-time qPCR analysis revealed that the mRNA expression of stemness-associated transcription factors—SOX2, CD133, and BMI1—was significantly increased in LNCaP cells due to *INMT* overexpression (Fig. 3C). Additionally, western blot assay was used to measure the protein expression levels of SOX2, BMI1, and CD133 in LNCaP cells, which were found to be upregulated by INMT. However, *INMT* overexpression did not cause an increase in KLF4 expression (Fig. 3D).

Endogenous INMT expression was knocked down by transient transfection of a INMT-specific shRNA vector. *INMT* silencing greatly attenuated ALDH activity (Fig. 3E and G) and reduced SOX2, BMI1, and CD133 expression in LNCaP and PC3 cells, although KLF4 was not altered after *INMT* silencing (Fig. 3F and H). The luciferase reporter assays indicated that INMT caused a 1.55- and 1.48-fold increase in SOX2 promoter (− 2546/+544) activity after transfection of PC3 and HEK293E cells with INMT plasmid for 48 h, respectively (Fig. 3J). Taken together, these results suggest that INMT promotes the stemness of PCa cells via SOX2.

### INMT expression is upregulated through the METTL3–m6A modification pathway in PCa cells

**Fig. 4:**
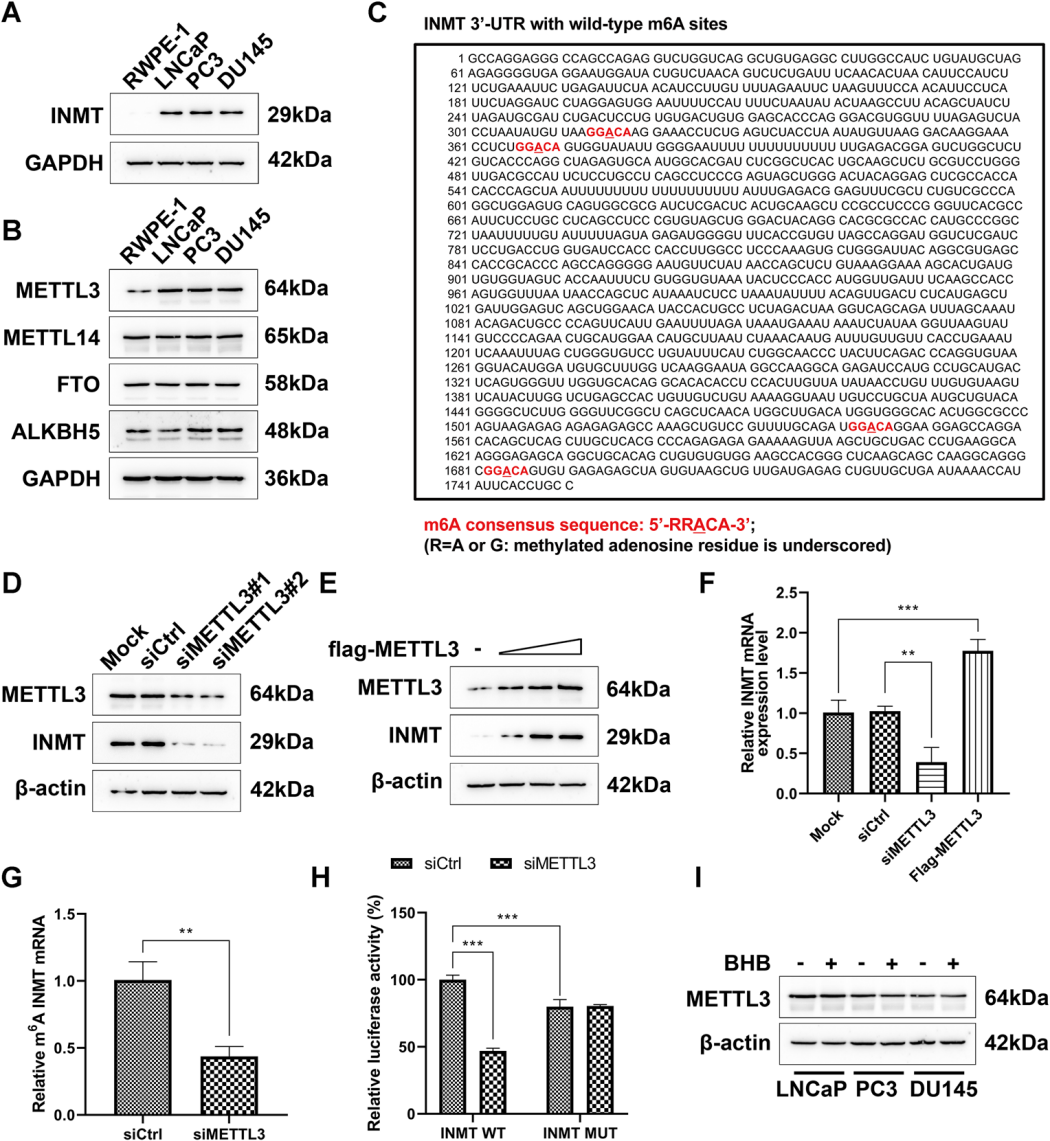
INMT expression is upregulated via the METTL3–m6A modification pathway in PCa cells. **A**, INMT expression levels in indicated cells were examined via western blot assay. **B**, The expression levels of m6A regulatory enzymes in corresponding cells were detected via western blot assay. **C**, Sequence analysis of m6A consensus sequences present on INMT 3’-UTR. **D**, INMT protein levels were detected via western blot assay after *METTL3* knockdown in LNCaP cells. **E**, INMT protein levels were detected via western blot assay after *METTL3* overexpression in LNCaP cells. LNCaP cells were transfected with various doses of METTL3 expression plasmids, followed by immunoblotting. **F**, Real-time qPCR was performed to detect the relative INMT mRNA levels after *METTL3* silencing or overexpression in LNCaP cells. **G**, m6A meRIP qRT–PCR assays were performed to determine changes in the levels of INMT mRNA undergoing m6A methylation modifications. **H**, Dual luciferase reporter assays were conducted in LNCaP cells transfected with WT or mutant (MUT) luciferase-INMT 3’-UTR reporter. Luciferase activity of INMT 3′-UTR was tested and normalized to renilla luciferase activity. **I**, The effects of BHB on the expression of METTL3 were detected via western blot assay in indicated cells. Paired and two-tailed Student’s t-tests were performed, and significant differences were designated as *p < 0.05, **p < 0.01, and ***p < 0.001

The m6A modification is one of PTM types, and PCa is often accompanied with upregulated METTL3 expression and aberrantly elevated m6A modification levels [39]. Our result showed that the expression levels of INMT were upregulated in the three PCa cell lines (Fig. 4A). Several enzymes, including METTL3, METTL14, ALKBH5, and FTO, involve the process of m6A modification [40]. Our study revealed that the expression levels of METTL3 were upregulated in LNCaP, PC3, and DU145 cells, whereas those of METTL14, ALKBH5, and FTO did not differ significantly among the cells (Fig. 4B). The SRAMP m6A forecast website (http://www.cuilab.cn/sramp) predicted that INMT 3’-UTR yielded four m6A consensus sequences (m6A-modified adenosine residues are underlined), which were using (Fig. 4C). Next, we investigated whether METTL3-induced m6A methylation modifications cause alterations in INMT expression. We revealed that siRNA-induced silencing of *METTL3* significantly reduced the protein expression level of INMT in LNCaP cells (Fig. 4D). In contrast, a dose-dependent increase in INMT protein levels was observed after *METTL3* overexpression in LNCaP cells (Fig. 4E). Consistently, the mRNA expression of INMT was downregulated by *METTL3* knockdown and upregulated by *METTL3* overexpression in LNCaP cells (Fig. 4F). We also measured the expression of methylated mRNA of INMT via m6A meRIP qRT–PCR. Total RNAs were immunoprecipitated with m6A antibodies, and the immunoprecipitated RNA was subjected to qRT–PCR to amplify the INMT 3’-UTR. *METTL3* silencing decreased the methylation of INMT mRNA, suggesting that INMT mRNA is indeed a target of METTL3-dependent m6A modifications (Fig. 4G).

To verify that INMT mRNA undergoes METTL3-dependent m6A methylation, we performed dual luciferase experiments, which indicated that *METTL3* knockdown significantly decreased the luciferase activity. By comparing WT and mutant reporters, we found that adenosine mutations in the identified consistent m6A sequences resulted in diminished luciferase activity in LNCaP cells (Fig. 4H). These results demonstrate that INMT expression is upregulated in PCa cells through the METTL3–m6A modification pathway. Treatment with BHB in LNCaP, PC3, and DU145 cells did not affect the expression levels of METTL3, indicating that the antagonistic effects of BHB on the malignant phenotype of PCa cells are not dependent on inhibition of the m6A modification pathway (Fig. 4I).

### BHB inhibits the malignant phenotypes of PCa via kbhb of INMT

**Fig. 5:**
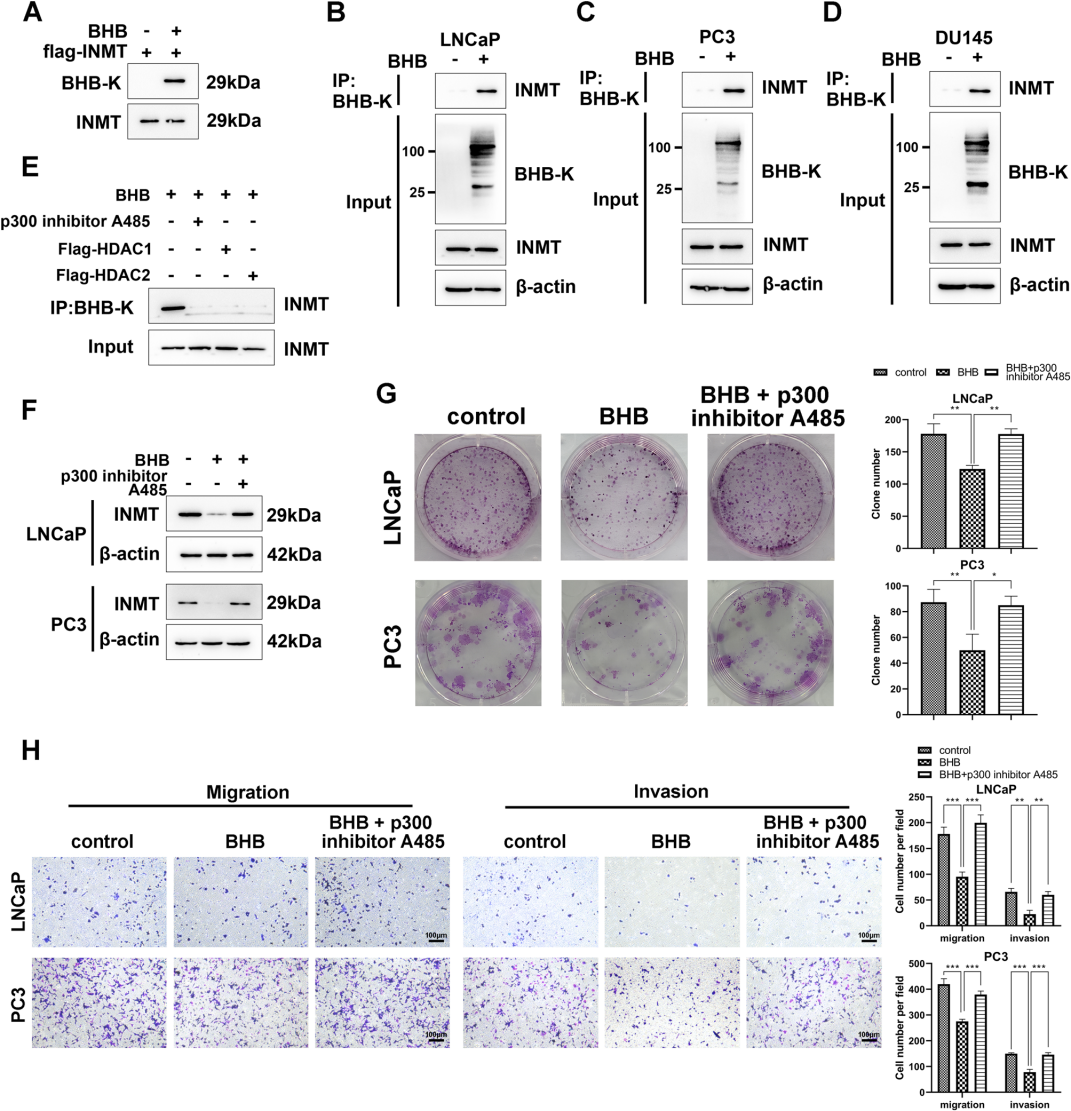
BHB suppresses malignant phenotypes of PCa via β-hydroxybutyrylation of INMT. **A**, Detection of kbhb modifications in 293T cells with *IMNT* overexpression. The Flag–INMT fusion proteins were purified from 293T cells treated with or without 15 mM BHB and detected via western blot assay using anti-INMT or anti-pan-β-hydroxybutyrylated lysine antibodies (BHB-K). **B**-**D**, kbhb modification of endogenous INMT in LNCaP, PC3, and DU145 cells. Cell lysates treated with or without 15 mM BHB were subjected to immunoprecipitation (IP) with BHB-K antibodies and then detected via western blot assay with INMT antibodies. Whole-cell lysates without IP were also detected via western blot assay with INMT, BHB-K, or actin antibodies as the input. **E**, Identification of enzymes regulating the kbhb of INMT via immunoprecipitation. Cell lysates with indicated treatment were immunoprecipitated (IP) with BHB-K antibodies and then detected via western blot assay with INMT antibodies. Input cell lysates were also subjected to INMT detection via western blot assay. **F**, Western blot analysis of the effects of BHB-mediated kbhb on INMT expression. Cell lysates treated with 15 mM BHB or 2.5 µM p300 inhibitor A485 were subjected to western blot assay with INMT and actin antibodies. **G**, Colony formation assays to assess the effects of BHB-mediated kbhb on in vitro tumor growth. **H**, Transwell assays were performed to evaluate the effects of BHB-mediated kbhb on migration and invasion of indicated cells. Experiments were performed in triplicate, and data were expressed as the mean ± standard deviation (SD). Paired and two-tailed Student’s t-tests were performed, and significant differences were designated as *p < 0.05, **p < 0.01, and ***p < 0.001

Since the above analysis revealed that the effect of BHB is independent on the m6A modification pathway, we supposed whether BHB regulates INMT via kbhb, other type of PTM. To identify kbhb-modified INMT proteins, we first purified and enriched Flag–INMT fusion protein in BHB-treated 293T cells transfected with Flag–INMT plasmid. Then, the fusion proteins were subjected to western blot assay with anti-INMT and anti-pan BHB-K. The results indicated that the enriched INMT could be identified by anti-INMT antibodies in all cells; however, it was only recognized by BHB-K antibody in cells treated with BHB, suggesting that INMT is modified by kbhb in the presence of BHB (Fig. 5A). Next, we assessed whether kbhb of endogenous INMT occurs in the three PCa cell lines—PC3, LNCaP, and DU145—showing the presence of wild-type INMT expression and intact INMT signaling pathway. The cell lysates from BHB-treated or untreated cells were immunoprecipitated with BHB-K antibodies and then immunoblotted with INMT antibodies. The results showed that the level of INMT in kbhb-enriched lysates from BHB-treated cells was significantly higher than that from untreated cells, suggesting that kbhb of endogenous INMT occurs via BHB (Fig. 5B and C, and 5D). To further investigate which enzyme is involved in kbhb of INMT, LNCaP cells were transfected with pFlag–HDAC1 or pFlag–HDAC2 and treated with BHB (15 mM) or the p300 inhibitor A485 (2.5 µM). The cell lysates were immunoprecipitated with BHB-K antibodies and then immunoblotted with INMT antibodies. The results in Fig. 5E showed that the group treated with BHB alone could detect the expression of INMT. p300 inhibitor A485, HDAC1, and HDAC2 inhibited the kbhb of INMT, suggesting that all three enzymes were involved in the kbhb of INMT. p300 exhibited kbhb transferase activity, whereas HDAC1 and HDAC2 were kbhb deacetylases, respectively (Fig. 5E).

To determine whether BHB-induced kbhb of INMT affects INMT expression, we treated LNCaP and PC3 cells with BHB (15 mM) and p300 inhibitor A485 (2.5 µM) for 48 h. Treatment with BHB alone attenuated INMT protein expression. In contrast, cotreatment with p300 inhibitor A485 reversed the reduction in INMT protein expression, suggesting that the kbhb of INMT by BHB inhibited INMT expression in PCa cells (Fig. 5F). We next investigated whether BHB-induced kbhb of INMT influences the promalignant phenotypic function of INMT. The proliferative capacity of LNCaP and PC3 cells was assessed via colony formation assay. The results indicated that BHB alone significantly inhibited the colony formation ability of malignant tumor cells. In contrast, cotreatment with A485, a p300 inhibitor that inhibits the kbhb of INMT, counteracted the effect of BHB (Fig. 5G). The migrative and invasive capacity of LNCaP and PC3 cells was also analyzed with or without BHB or A485 treatment. Similarly, inhibition of cell migration and invasion by BHB was reversed when cells were cotreated with A485, an inhibitor of INMT kbhb (Fig. 5H). Altogether, our findings suggest that BHB inhibits the malignant phenotypes of PCa via kbhb of INMT.

### BHB attenuates the growth and metastasis of LNCaP tumors in xenograft nude mouse models

**Fig. 6:**
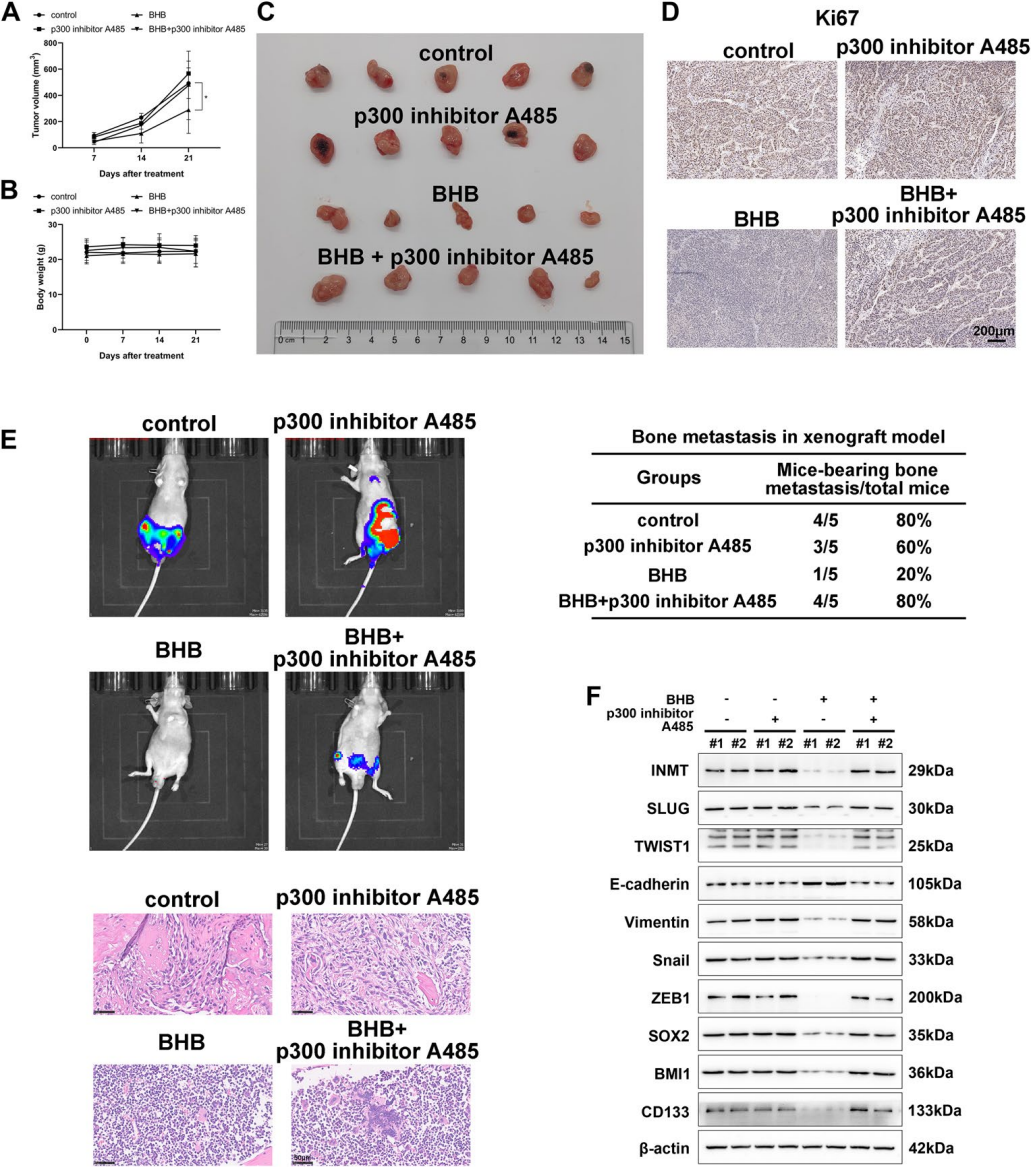
BHB attenuates the growth and metastasis of LNCaP tumors transplanted in xenografted nude mice. **A**, Growth curves of subcutaneous xenografts of LNCaP cells. Nude mice bearing xenograft tumors were treated with BHB (100 mg/kg) or p300 inhibitor A485 (30 mg/kg) for 25 days. The measured tumor volumes versus time were plotted. **B**, The body weights of mice in various treatment groups were detected. **C**, Weight of tumors dissected on day 25 after treatment or no treatment. Representative tumor images and statistical charts are shown. **D**, **H**&**E** staining and immunohistochemistry for Ki-67 antigen was performed in xenograft tumors from mice (40×). Scale bars, 100 μm. **E**, Incidence of metastasis in the bone of mice after BHB treatment or no treatment. Representative images of mouse imaging in vivo and **H**&**E** staining in different treatment groups are shown. **F**, Western blot analysis of CSCs and EMT-associated markers in xenograft tumors from mice on day 25. Data were expressed as the mean ± standard deviation (SD). Paired and two-tailed Student’s t-tests were performed, and significant differences were designated as *p < 0.05, **p < 0.01, and ***p < 0.001

The in vivo effect of BHB on PCa cells was assessed in detail by establishing a xenograft nude mouse model transplanted with LNCaP cells. BHB (100 mg/kg) and p300 inhibitor A485 (30 mg/kg) were not significantly toxic at this dose, as there were no differences in locomotion, feeding behavior, or mortality in any group (data not shown), and the weight of the mice remained stable and did not differ in either treatment group (Fig. 6B).

Analysis of tumor growth curves revealed that BHB significantly attenuated the growth of LNCaP tumors. In contrast, after cotreatment with A485, their growth rate returned to a level similar to that of the control group (Fig. 6A). In addition, the tumors treated with BHB were smaller and lighter than those in the control group. In contrast, cotreatment with A485 counteracted the effect of BHB (Fig. 6C). Immunohistochemistry for Ki-67 antigen (an indicator of cell proliferative capacity) showed that Ki67 expression was lower in the BHB treatment group, whereas the expression was increased again in the A485 cotreatment group (Fig. 6D). The in vivo imaging system showed that the fluorescence signal (representing bone metastasis) of BHB treatment group was decreased compared with that of the control group. The fluorescence signal returned to the control level after combined treatment with A485. Compared with the control group (incidence of bone metastases: 4/5, 80%), mice treated with BHB showed a 75% reduction in the incidence of bone metastases (1/5, 20%), whereas the incidence of bone metastases increased again after cotreatment with A485 (4/5, 80%) (Fig. 6E). Immunoblotting of mouse xenograft tissues demonstrated that BHB potently suppressed the levels of stemness- and EMT-related factors, and their expression was reversed to a higher level after cotreatment with A485 (Fig. 6F). All these results emphasize the anti-PCa effect of BHB in vivo and the role of kbhb mechanism in the antagonistic effect of BHB against PCa.

## Discussion

Although the specific role of BHB, a ketone body, is still not well understood, emerging evidence suggests its important role in tumor biology. According to a previous report, BHB can reduce the proliferative capacity of colonic crypt cells and potently inhibit intestinal tumor growth in mouse models and organoids [13]. In addition, BHB-induced metabolic reprogramming attenuated the growth of pancreatic cancer and cachexia in xenograft mouse models [11]. Importantly, BHB has a significant inhibitory effect on cancer metastasis, as evidenced by an inhibitory effect on the highly metastatic properties of VM-M3 cells and prolonged survival in VM-M3 xenograft mice [12]. However, many researchers have also expressed opposite views regarding the effect of BHB on tumors. BHB has been found to increase the “stemness” of cancer cells, driving growth, metastasis, recurrence, and poor clinical outcomes in breast cancer [17, 41]. In addition, BHB acts as a cellular endogenous or systemic fuel to promote the growth and progression of pancreatic ductal adenocarcinoma [18]. Thus, it remains unclear whether the effects of BHB on cancer are related to the type of cancer and the underlying mechanisms. Herein, we validated the antimalignant phenotype effect of BHB on PCa using three PCa cell lines in vitro and xenograft mouse models in vivo. Our study is valuable as it reveals new diet-induced endogenous mechanisms that inhibit prostate tumor growth.

CSCs represent a subpopulation within tumors with the potential for self-renewal and nondirectional differentiation [42]. It drives tumor growth and sows the seeds of metastasis; moreover, it is closely related to therapeutic resistance and relapse of many tumor types, including PCa [43–45]. Therefore, stem cell properties have emerged as a target for cancer therapy. ALDH, SOX2, BMI1, CD133, and KLF4 have previously been used as stemness-associated markers of prostate CSCs [46–49]. Several studies have indicated a potential role of BHB in inhibiting the stemness of CSCs. BHB was reported to significantly reduce the proportion of CD133 + A2780CP cells (an ovarian cancer cell line) [50]. In addition, BHB exhibited inhibition of proliferative capacity and stemness of glioma stem-like cells by disrupting metabolic homeostasis and mitochondrial function [8]. Consistent with these studies, our investigations showed that BHB significantly inhibited the stem-like properties of PCa cells. Tumor sphere formation, ALDH activity, and the expression levels of stemness-related factors (SOX2, BMI1, and CD133) in PCa cells were potently reduced after BHB treatment. EMT is an important process governing the characteristic features of CSCs. For example, ZEB1 promotes the migration of CSCs by suppressing repressive stemness microRNAs in pancreatic cancer cells [51]. TWIST1 upregulates BMI1, a marker of CSCs that is essential for tumor initiation capacity in head and neck squamous cell carcinomas [52]. Elevated SLUG expression in breast tumors induces overexpression of stem-like genes, such as *BMI1* and *CD133*[53]. Therefore, we speculate that the potential mechanisms of the inhibitory effects of BHB on the stemness of PCa cells may be related to our finding that BHB inhibits the process of EMT.

INMT is a newly emerging molecule that has gained considerable interest; however, its role in cancer remains obscure. INMT expression has been reported to be dysregulated in lung cancer, meningioma, and PCa [31, 54–56]. Our study revealed that INMT expression was upregulated in PCa cells, and this upregulation was mediated via the METTL3–m6A pathway. m6A modification, one of the epigenetic regulatory mechanisms, modulates gene expression and function by regulating many aspects of RNA biology, such as pre-mRNA splicing, nuclear transport, subcellular localization, RNA decay, and translation ability [57]. As a reversible epitranscriptome modulator, METTL3 is highly expressed in PCa and is essential for the proliferation and metastasis of various PCa cell lines [39, 58, 59]. Our results consistently showed upregulation of METTL3 in three PCa cell lines and its role in the suppression of INMT mRNA decay. Recent studies have reported conflicting views on the effects of INMT on PCa, with one study suggesting that INMT plays a tumor-suppressive role, whereas the other suggesting that INMT promotes PCa development and castration resistance [30, 31]. Our results demonstrate that INMT is an oncogenic gene that promotes the stemness characteristics of PCa cells. This enhancement in stem-like properties may be related to the improvement of SOX2 activity. The transcription factor SOX2, which induces pluripotency, is an important embryonic stem cell transcriptional factor capable of inducing cellular reprogramming. SOX2 endows cells with CSC characteristics and a malignant, aggressive phenotype during PCa development [60]. Our study revealed that INMT can enhance the promoter activity of SOX2.

Previous studies have found that METTL3 is upregulated in PCa and can promote PCa growth and metastasis [39, 59]. However, the observed anticancer effect of BHB was not achieved through the METTL3–m6A pathway, as BHB did not affect the expression of METTL3. kbhb, a recently discovered and evolutionarily conserved PTM, is driven by BHB and plays a pivotal role in cell function [32, 33]. The results of mass spectrometry analysis in a previous study and the data from immunoprecipitation assay in our study suggest that INMT may be β-hydroxybutyrylated [34]. The lysine kbhb of INMT decreased the INMT expression in PCa. Our study revealed a novel finding that BHB exerts its anticancer effects through INMT kbhb, and in vivo experiments also confirmed that the antitumor effect of BHB is diminished when kbhb is inhibited. However, there are some limitations in this study. First, the mechanism underlying the decrease in INMT expression levels after the occurrence of kbhb in INMT warrants in-depth exploration. Besides, the present study only selected the LNCaP cell line as the Xenograft tumor model, which may not rule out the cell-specific effect. Hence, and we will establish a Xenograft tumor model with other cell lines for further validation in the future.

In summary, our results demonstrated that INMT, an oncogenic gene in PCa, is highly expressed in PCa cells via the METTL3–m6A pathway and can promote stem-like properties of these cells. In contrast, BHB, an endogenous ketone of the body, can exert an anti-PCa malignant phenotype by driving INMT kbhb. Our study revealed a novel molecular mechanism that provides a theoretical basis for BHB to become a new alternative for cancer treatment in addition to surgery, chemotherapy, and immunotherapy. However, it remains unclear whether BHB inhibits PCa stemness through multiple pathways and the mechanism by which kbhb regulates INMT expression levels. These details need to be explored further in the future.

### Electronic supplementary material

Below is the link to the electronic supplementary material.

Supplementary Material 1

## Data Availability

The datasets used and/or analysed during the current study are available from the corresponding author on reasonable request.
