# Supplementary material for: β-hydroxybutyrate inhibits malignant phenotypes of prostate cancer cells through β-hydroxybutyrylation of indoleacetamide-N-methyltransferase
Source: Cancer Cell Int. 2024 Mar 30;24:121. doi: 10.1186/s12935-024-03277-6 (PMC10981303; doi:10.1186/s12935-024-03277-6)
Supplement: Supplementary file 1 — Supplementary Material 1 [file 12935_2024_3277_MOESM1_ESM.doc]

Supplemental Table S1: The sequences of primers used in this study

| Genes | Primers | Sequences |
| --- | --- | --- |
| INMT | Forward | 5’-TGGAGAAAGAGGAGGTGGAGCAG-3’ |
|  | Reverse | 5’-GGCAGCATTGGTGACAGAGTAGC-3’ |
| SOX2 | Forward | 5’-AATGCCTTCATGGTGTGGT-3’ |
|  | Reverse | 5’-CTTCTCCGTCTCCGACAAA-3’ |
| BMI1 | Forward | 5’-CTGATGTGTGTGCTTTGTGG-3’ |
|  | Reverse | 5’-TGGTCTCCAGGTAACGAACA-3’ |
| PROM1(CD133) | Forward | 5’-CAGTCTGACCAGCGTGAAAA-3’ |
|  | Reverse | 5’-GGATTGATAGCCCTGTTGGA-3’ |
| GAPDH | Forward | 5’-CATGACCACAGTCCATGCCAT-3’ |
|  | Reverse | 5’-AAGGCCATGCCAGTGAGCTTC-3’ |
|  |  |  |
